# Molecular Characterisation and Antimicrobial Resistance Patterns of *Listeria monocytogenes* From Dairy Sources

**DOI:** 10.1002/vms3.71265

**Published:** 2026-09-30

**Authors:** Mohammad Reza Heidari, Amir Shakerian, Reza Sharafati Chaleshtori, Ebrahim Rahimi

**Affiliations:** ^1^ Department of Food Hygiene ShK.C. Islamic Azad University Shahrekord Iran; ^2^ Research Center of Nutrition and Organic Products (R.C.N.O.P) ShK.C. Islamic Azad University Shahrekord Iran; ^3^ Research Center for Biochemistry and Nutrition in Metabolic Diseases Kashan University of Medical Sciences Kashan Iran; ^4^ Department of Veterinary ShK.C. Islamic Azad University Shahrekord Iran

**Keywords:** antibiotic resistance, *listeria monocytogenes*, listeriosis, milk and dairy products

## Abstract

**Introduction and Aim:**

*Listeria monocytogenes* (*L. monocytogenes*) is a major food pathogen that causes severe infections, especially in the susceptible population. Dairy products and milk are some of the possible vehicles through which it can be transmitted both as a threat to the health of people and as an economic issue. The study conducted was to explore the prevalence, virulence characteristics, and antimicrobial resistance patterns of *L. monocytogenes* along the supply chain of milk in Yazd Province, Iran.

**Material and Method:**

Collection of 370 samples (raw milk, pasteurised milk, dairy equipment, transport containers and hands and gowns of workers) was done between 2022 and 2023. Identification and isolation of *L. monocytogenes* was done using standard biochemical, bacteriological and molecular tests. The assessment of antimicrobial susceptibility was done using the Kirby‐Bauer disk diffusion, and the multi‐antibiotic resistance (MAR) index was determined. The presence of important virulence genes and resistance determinants was identified by PCR.

**Results:**

Overall, 10 isolates (2.7%) were confirmed as *L. monocytogenes*. Raw milk had the highest contamination rate (5.29%), while pasteurised milk and worker samples were negative. High‐level resistance was observed against nalidixic acid, penicillin G, ceftazidime (100%), cefepime and ceftriaxone (90%), whereas first‐line clinical drugs such as gentamicin, amikacin, levofloxacin, vancomycin, trimethoprim‐sulfamethoxazole and erythromycin showed full susceptibility. Virulence genes *prfA, inlA* and *iap* were universally present, with high prevalence of resistance genes including *lnuA/B, ermA/B* and *tetA/M*.

**Conclusion:**

The results suggest that raw milk may be a significant reservoir of multi‐drug‐resistant *L. monocytogenes* in this region and the studied supply chain. Therefore, hygiene measures and continuous monitoring are key to preventing contamination and protecting public health.

## Graphical abstract

This study presents a comprehensive surveillance of *L. monocytogenes* along the dairy supply chain, from raw milk collection to final products and environmental sources. The methodological framework encompasses conventional microbiological isolation and identification, followed by phenotypic antimicrobial susceptibility testing and calculation of the multiple antibiotic resistance (MAR) index. The assessment is further deepened by molecular characterisation using PCR to detect key virulence markers and antibiotic resistance genes, ultimately evaluating the efficacy of thermal processing and hygiene practices in the dairy industry.

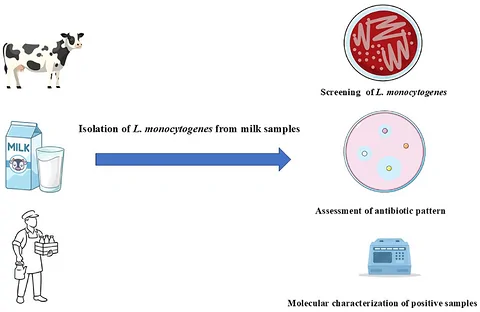

## 1 Introduction

The problem of food‐borne diseases remains worldwide, and, as a result, technologies must be introduced, and close monitoring of the food chain should be made to ensure the quality and safety of products (Dabija et al. 2025). The milk and dairy items are universal products, which provide high‐quality proteins, vitamins and valuable minerals to the human diet (Holzhauer and Wennink 2023). The dairy production can have a great improvement in the local, regional and national economies, as well as providing employment and producing cash, which are critical determinants regarding health (Al‐Noman et al. 2022). However, a number of potential health issues associated with dairy production and consumption have been identified, including food‐related and occupational risk, chronic diet disorders such as milk allergy, environmental changes and zoonotic diseases (Holzhauer and Wennink 2023). Milk is a major pathway of the zoonotic diseases and usually becomes contaminated either directly or indirectly by contact with the infected animals. The bacteria may get into the milk stock through the mastitic mammary glands, or most commonly through faecal contamination during the milking process (Pradhan and Karanth 2023). Furthermore, environmental conditions on the dairy farms, like silages, soils and water contain contaminated materials that are reservoirs, enhancing the transmission of these diseases out of the animal environment into the raw milk supply chain (Kapoor et al. 2023).

*Listeria monocytogenes* (*L. monocytogenes*) is a pathogenic food‐borne microorganism which is defined by the capacity to withstand severe environmental conditions such as low temperature and salt content (Li et al. 2024). This Gram‐positive and intracellular organism causes Listeriosis, a severe disease with a high rate of hospitalisation and mortality especially in the vulnerable groups of patients such as infants, elderly and immunocompromised patients (Obaidat 2024). Its capacity to form resilient biofilms on food‐contact surfaces and machinery can complicate hygiene management in some of the food‐processing facilities of the dairy industry (Bagatella et al. 2022). The virulence factors that the *L. monocytogenes* control its pathogenicity are a group of virulence factors, mainly controlled by the *prfA* gene, the primary transcriptional regulator of the expression of *Listeria* Pathogenicity Island 1 (LIPI‐1) (Sibanda and Buys 2022). The key components of this regulatory network are the gene of *listeriolysin O* (key in avoiding the phagosome of the host cell), the *internalins* (*InlA* and *InlB*) and the actin‐polymerisation protein (*ActA*) (Quereda et al. 2021). Recent genomic studies have revealed that *prfA* variability and *hly* gene presence have a direct effect on haemolytic activity and general case severity of clinical listeriosis (Quereda et al. 2021). The understanding of the distribution of these specific genetic markers in isolates of milk, equipment, and hands of workers is important to assess the true threat of public health. Since antibiotics are the mainstay of treatment for listeriosis, the emergence of antimicrobial resistance poses a significant threat to the treatment of the disease.

Besides, the development of antimicrobial resistance (AMR) in *Listeria* species has complicated clinical treatment. Due to the wide use of antimicrobial medications in veterinary medicine, the development of antibiotic‐resistant microbes has occurred, regardless of their past vulnerability to various medicines (Díaz‐Martínez et al. 2025). Monitoring activities have shown an increasing trend in resistance to first line treatment like ampicillin. Food safety management is hampered by the prevalence of multi‐drug resistant (MDR) strains in the dairy setting, which is usually a result of poor cross‐contamination through the hands of workers or through contaminated equipment. The molecular distribution and antimicrobial resistance of *L. monocytogenes* throughout the dairy value chain, especially in developing regions, are poorly known, despite the known risk of listeriosis. Currently, there is no way to follow the pathogen from its point of region into the processing operation and the end consumer, resulting in an important means of failure for implementing targeted food safety interventions. So, the aim of the present study was to fill this gap by evaluating the prevalence, virulence factors, and antibiotic resistance patterns of raw and pasteurised milk, dairy processing equipment, and workers' hands associated with *L. monocytogenes* in Yazd province, Iran.

## 2 Material and Methods

### 2.1 Sample Collection

The study is an experimental, descriptive, cross‐sectional laboratory study that was conducted in Yazd Province, Iran, from 2022 to 2023. Sampling was conducted in both warm and cold seasons to evaluate environmental and temperature changes in bacterial prevalence. A total of 370 samples were collected using the simple random sampling technique; they were raw milk collected from dairy cooperatives and collection centres (n = 170), pasteurised milk, various commercially branded milks (n = 90), milking equipment and transport containers (n = 60), and the hands and gowns of the workers in the milking and collection facilities (n = 50). Raw milk samples were collected in sterile plastic bowls twice a day, morning and evening, to obtain a comprehensive assessment of microbial load. The samples were collected from dairy farms following traditional and semi‐industrial management rules, where cows were primarily kept in open‐shed barns with standardised feeding protocols. Pasteurised milk was also provided in its commercial packaging at retail stores. The pasteurised milk samples were collected from local commercial dairies that use standard high‐temperature, short‐time (HTST) pasteurisation protocols (72°C–75°C for 15 s) before packaging. Sterile swabs were used to surface sample the machinery, milk churns and hands and clothes of workers and put into screw‐capped bottles filled with buffered peptone water (BPW). All the samples were shipped to the laboratory under extreme cold chain conditions in an insulated cold box. The inclusion criteria required that samples should be received within the stipulated time and temperature and free of leakages and external contamination; however, samples that were subject to sunlight, with signs of thermal maltreatment or were having a broken packing or tubes were disqualified in the study.

### 2.2 Isolation and Detection of *L. monocytogenes*

Isolation and identification of *L. monocytogenes* were done in a procedure that had been standardised by the U.S. Food and Drug Administration (FDA). For initial enrichment, 25 mL of milk samples, equipment milk samples and swabs were pre‐enriched in Buffered Peptone Water (BPW) and incubated at 37°C. Afterward, 25 mL of the pre‐enriched solution was placed in 225 mL of *Listeria* Enrichment Broth (LEB, ibresco, Iran) and incubated at 30°C for 48 h. A portion of the improved broth was loop streaked on Palcam Agar (Condalab, Spain) and Chromogenic *Listeria* Agar (Condalab, Spain) to obtain selective colonies and incubated at 37°C to obtain pure, isolated colonies. The putative *Listeria* colonies on Palcam Agar were brownish‐green with a clear black halo and a small central depression. The identification was done by Gram staining, and the organism was Gram‐positive short rods or coccobacilli, and then the organism was subjected to multiple biochemical tests. The tests were catalase, oxidase, Methyl Red‐Voges Proskauer (MR‐VP), and sugar fermentation tests (rhamnose and xylose). Motility was measured at 25°C and 37°C. Species identification was also done by beta‐haemolysis of sheep blood agar. *L. monocytogenes* ATCC13932 was used as a positive control, and *Staphylococcus aureus* ATCC12600 was used as a negative control to confirm biochemical and haemolysis tests (Kayode and Okoh 2022).

### 2.3 Antibiotic Susceptibility Testing

The antimicrobial resistance of the isolates was evaluated by performing the Kirby‐Bauer disk diffusion test using the recommendations of the Clinical and Laboratory Standards Institute (CLSI, M45, 3r). Bacterial suspensions were made in sterile saline to give a 0.5 McFarland turbidity standard and incubated onto Muller‐Hinton agar (MHA, Condalab, Spain) by using sterile swabs. The panel of antibiotics disks was used, including ampicillin (AMP, 10 µg), gentamicin (GM, 10 µg), ciprofloxacin (CIP, 5 µg), levofloxacin (LEV, 5 µg), vancomycin (VA, 30 µg), chloramphenicol (C, 30 µg), ceftazidime (CAZ, 10 µg), penicillin G (PEN, 10 µg), imipenem (IPM, 10 µg), ampicillin‐sulbactam (A/S, 30 µg), ceftriaxone (CRO, 30 µg), cefepime (CPM, 30 µg), amikacin (AM, 10 µg), nalidixic acid (NA, 30 µg), erythromycin (E, 15 µg), clindamycin (CD, 2 µg), tetracycline (TE, 30 µg), and trimethoprim‐sulfamethoxazole (SXT, 25 µg). The areas of the zones of inhibition were measured using a graduated ruler or a calliper after 24 h of incubation at 37°C. The analysis was done with the help of the CLSI reference tables to categorise the isolates into susceptible, intermediate or resistant to either antimicrobial agent. The multiple antibiotic resistance index (MAR) was determined for each *L. monocytogenes* isolate. To measure the MAR index, we used the formula below: MAR: a/b that a is the number of antibiotics to which the isolate was resistant and b is the total number of antibiotics tested. Isolates having MAR index greater than 0.2 were regarded to be of high‐risk sources with a high exposure to antibiotics.

### 2.4 DNA Extraction

A single colony of *L. monocytogenes* was picked on the Tryptic Soy Agar (TSA, ibresco, Iran) and allowed to grow overnight at 37°C. One colony was inoculated in 5 mL of Tryptone Soy Broth (TSB, ibresco, Iran) and incubated at 37°C in aerobic conditions for 18–24 h. After that, the overnight culture was centrifuged at 4000 × g for 10 min. A bacterial pellet was washed in 1 mL of sterile distilled water, followed by the resuspension of the bacterial pellet in 400 µL of phosphate‐buffered saline (PBS; Aminsan, Iran). According to the instructions provided by the manufacturer, genomic DNA was extracted using a commercial Parstous DNA extraction kit (Iran). The DNA was stored at −20°C to be subjected to further molecular examination.

### 2.5 Detection of Virulence and Antimicrobial Resistance Genes

Polymerase chain reaction (PCR) was used to detect the presence of virulence and antibiotic resistance‐associated genes in isolates of *L. monocytogenes*. The pathogenic potential of the isolates was assessed by examining five major virulence genes, including *hlyA*, *inlA*, *inlB*, *prfA* and *iap*. Gene‐specific primers were used to analyse *aadB*, *lnuB*, *lnuA*, *ermA*, *ermB*, *tetA* and genes *tetM*. The total of the reaction volume was 25 µL including 12 µL of 2x RED Master Mix (Ampliqon, Denmark), 5 µL of template DNA, 1 µL of forward and reverse primers, and 7 µL of nuclease‐free water. The conditions used to amplify DNA were an initial denaturation stage (10 min at 95°C), followed by denaturation (30 s at 95°C), primer‐specific annealing (30 s at 55‐65°C) and extension (30 s at 72°C). In order to ensure high specificity, all PCR reactions were performed separately for each gene. Also, the temperature condition for each set of primers were optimised based on the annealing temperature listed in Table 1. DNA of *L. monocytogenes* ATCC13932 was used as a positive control, and DEPC water was used as a negative control. Agarose gel of 2% PCR products was run through the electrophoresis process and stained with safe DNA dye and viewed under UV light.

**TABLE 1:** List and details of the primers used in the present study.

Gene name	Sequence (5ˈ to 3ˈ)	Annealing temperature (°C)	Product size	Ref.
*hlyA*	F: GCATCTGCATTCAATAAAGA R: TGTCACTGCATCTCCGTGGT	50	174	(El‐Zamkan et al. 2021)
*inlA*	F: ACGAGTAACGGGACAAATGC R: CCCGACAGTGGTGCTAGATT	55	800	(El‐Zamkan et al. 2021)
*inlB*	F: AAAGCACGATTTCATGGGAG R: ACATAGCCTTGTTTGGTCGG	57	146	(Mureddu et al. 2014)
*prfA*	F: TCTCCGAGCAACCTCGGAACC R: TGGATTGACAAAATGGAACA	50	1092	(El‐Zamkan et al. 2021)
*iap*	F: ACAAGCTGCACCTGTTGCAG R: TGACAGCGTGTGTAGTAGCA	56	131	(Chen et al. 2025)
*aadB*	F: GAGCGAAATCTGCCGCTCTTG R: CTGTTACAACGGACTGGCCGC	57	310	(Wiśniewski et al. 2022)
*lnuA*	F: GGTGGCTGGGGGGTAGATGTATTAACTGG R: GCTTCTTTTGAAATACATGGTATTTTTCGATC	59	323	(Wiśniewski et al. 2022)
*lnuB*	F: CCTACCTATTGTTTGTGGAA R: ATAACGTTACTCTCCTATTC	54	405	(Wiśniewski et al. 2022)
*Erm A*	F: TCTAAAAAGCATGTAAAAGAA R: CTTCGATAGTTTATTAATATTAGT	60	645	(Agostinho Davanzo et al. 2021)
*Erm B*	F: GAAAAGGTACTCAACCAAATA R: AGTAACGGTACTTAAATTGTTTAC	60	639	(Agostinho Davanzo et al. 2021)
*tetM*	F: GTGGACAAAGGTACAACGAG R: CGGTAAAGTTCGTCACACAC	60	450	(Agostinho Davanzo et al. 2021)
*tetA*	F: GTTAAATAGTGTTCTTGGAG R: CTAAGATATGGCTCTAACAA	59	700	(Agostinho Davanzo et al. 2021)

### 2.6 Statistical Analysis

The statistical examination was carried out using SPSS 19.0 software. The chi‐squared test was used, with *p* < 0.05 indicating statistical significance.

## 3 Results

### 3.1 Prevalence of *L. monocytogenes* in Milk Chain

Out of the 370 samples isolated in the dairy chain, Yazd, 10 (2.7%) of the isolates were confirmed to be *L. monocytogenes* by molecular and biochemical techniques (Table 2). The transmission of the pathogen varied significantly in the case of different samples. Raw milk had the highest number of *L. monocytogenes* positive samples, with 9 out of 170 (5.29%) cases. In contrast, *L. monocytogenes* was identified in just 1 out of 60 (1.66%) samples collected from milking apparatus and transit containers. It is important to note that *L. monocytogenes* was not found in any of the pasteurised milk samples (0/90, 0%) or the samples from workers' hands and gowns (0/50, 0%). The data indicate that the major reservoir of this pathogen in the discussed region is raw milk.

**TABLE 2:** The frequency and prevalence of *L. monocytogenes* isolated from raw milk and dairy cooperatives in Yazd Province, Iran.

Sample type	Total number	Positive number	Percentage of positive samples
Raw milk	170	9	5.29
Pasteurised milk	90	0	0
Milking equipment	60	1	1.66
Workers' hands/gowns	50	0	0
Total	370	10	2.7

### 3.2 Frequency of *L. monocytogenes* in Raw Milk

In a study of 10 raw milk collection centres and dairy cooperatives in the Province of Yazd, it was established that 80 percent (8 out of 10) of them were contaminated with *L. monocytogenes* (Table 3). The positivity rate was highest in the cooperative Company B and cooperative Company J, where 10 percent of their samples were positive. Moderate levels of contamination were found in cooperative Company A, cooperative Company C, cooperative Company D, cooperative Company E, and cooperative Company G with a 5% transmission rate as each. But the samples of the cooperative Company F and cooperative Company H had no *L. monocytogenes* (0%). These data show that *L. monocytogenes* exists throughout the supply chain of raw milk in the region, albeit to a low extent.

**TABLE 3:** Distribution of *L. monocytogenes* isolated from various raw milk collection centres and cooperatives in Yazd Province, Iran.

Raw milk collection centres	Number of collected samples	Positive samples	Percentage of positive cases in each centre	Percentage of positive cases out of 170 samples
Cooperative Company A	20	1	5	0.58
Cooperative Company B	20	2	10	1.17
Cooperative Company C	20	1	5	0.58
Cooperative Company D	20	1	5	0.58
Cooperative Company E	20	1	5	0.58
Cooperative Company F	20	0	0	0
Cooperative Company G	20	1	5	0.58
Cooperative Company H	10	0	0	0
Cooperative Company I	10	1	10	0.58
Cooperative Company J	10	1	10	0.58
Total	170	9	—	—

### 3.3 Antibiotic Susceptibility Profile

Antimicrobial susceptibility testing of 10 *L. monocytogenes* strains isolated from raw milk showed different resistance patterns to different classes of antibiotics (Figure 1). As expected, *L. monocytogenes* showed relatively high intrinsic resistance against nalidixic acid (100%), ceftazidime (100%), penicillin G (100%), cefepime (90%) and ceftriaxone (90%). Also, the isolates were very sensitive to a number of clinically relevant agents, such as gentamicin (100%), amikacin (100%), levofloxacin (100%), vancomycin (100%), trimethoprim‐sulfamethoxazole (100%), chloramphenicol (100%), ciprofloxacin (100%), ampicillin‐sulbactam (100%) and erythromycin (100%). In addition, there was a high susceptibility of 90% with clindamycin. Imipenem had medium susceptibility and 50% of the isolates were resistant, and the remaining 50% had intermediate susceptibility. Resistance to tetracycline was observed in 30% (3/10) of the total *L. monocytogenes* isolates, while 20% (2/10) exhibited intermediate susceptibility to ampicillin. These data showed that although first‐line treatments are still very successful in the region, the detection of acquired resistance to imipenem and tetracycline requires close monitoring in the future.

**FIGURE 1:**
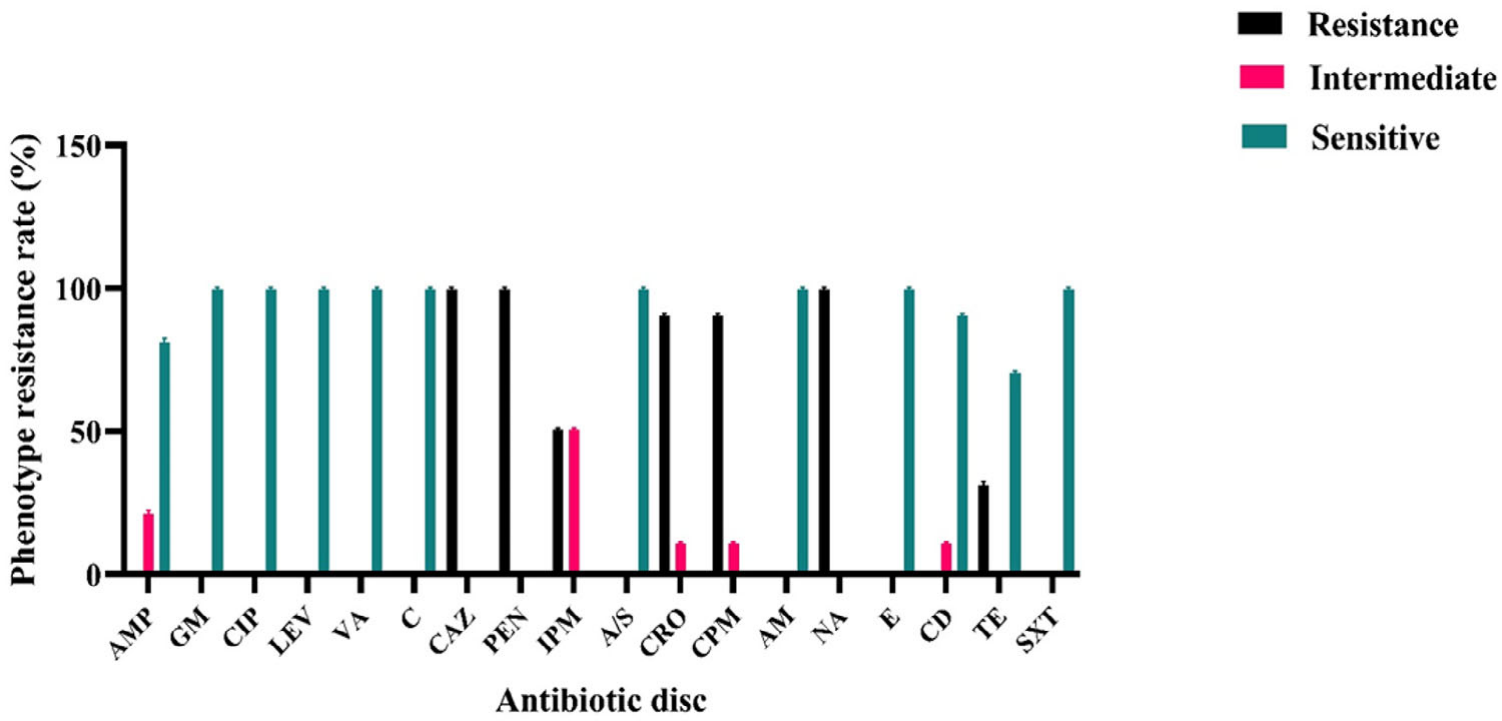
The antibiotic resistance patterns of *L. monocytogenes* strains isolated from raw milk and milking equipment.

### 3.4 Determination of the Multiple Antibiotic Resistance Index (MAR)

Due to the inherent resistance of *L. monocytogenes* to cephalosporins and nalidixic acid and to avoid unrealistic scores, multiple resistance index (MAR) values were calculated for 10 *L. monocytogenes* isolates based on 14 antibiotics. The range of MAR levels of *L. monocytogenes* isolates was between 0.07 and 0.21 (Table 4). It is important to note that 2/10 isolates had a value of the MAR index exceeding 0.2, which is characteristic of those that were found to be in high‐risk environments where antibiotics were used frequently. The largest MAR values were found in isolates with resistance to imipenem, clindamycin and tetracycline, and it indicates the evidence of multidrug‐resistant isolates in the dairy production chain.

**TABLE 4:** MAR index of *L. monocytogenes* strains isolated from raw milk and milking equipment.

Number of isolates	Number of antibiotics tested (b)	Number of resistant antibiotics (a)	MAR index (a/b)	Resistance pattern
1	14	3	0.21	IPM, CD, TE
2	14	2	0.14	IPM, CD
3	14	1	0.07	CD
4	14	3	0.21	IPM, CD, TE
5	14	1	0.07	CD
6	14	2	0.14	IPM, CD
7	14	2	0.14	CD, TE
8	14	1	0.07	CD
9	14	1	0.07	CD
10	14	1	0.07	IPM

### 3.5 Investigation of Virulence and Antibiotic Resistance Genes

The major virulence‐associated genes *prfA*, *inlA* and *iap* (100%) were present in all *L. monocytogenes* isolates, which means that these bacteria had a high pathogenic potential. The expression of the major pathogenicity factors is regulated by the *prfA* gene, which is the central regulator of *Listeria* virulence. The *inlA* and *inlB* genes, which lead to invasion of host cells, were identified in 100% and 90% of the isolates, respectively. Isolates were also found to have a high virulence capacity by the *hlyA* (90%) gene, which encodes listeriolysin O and is required to escape the phagosome (Figure 2A). On the determinants of antimicrobial resistance, the *lnuA* (90%) and the *lnuB* (80%) genes that are linked with resistance to lincosamide antibiotics like clindamycin were very common. *ErmA* (40%) and *ermB* (30%) genes that confer resistance to erythromycin, macrolides, lincosamides and streptogramin B were also found. Also, *aadB* gene (30%), associated with aminoglycoside resistance, and the tetracycline resistance genes *tetM* and *tetA* (30% and 30%, respectively), which cause ribosomal protection and efflux, respectively, were determined (Figure 2B). The coexistence of virulence and resistance genes is high, which points to the presence of possibly pathogenic and multidrug‐resistant strains of *L. monocytogenes* in the dairy environment.

**FIGURE 2:**
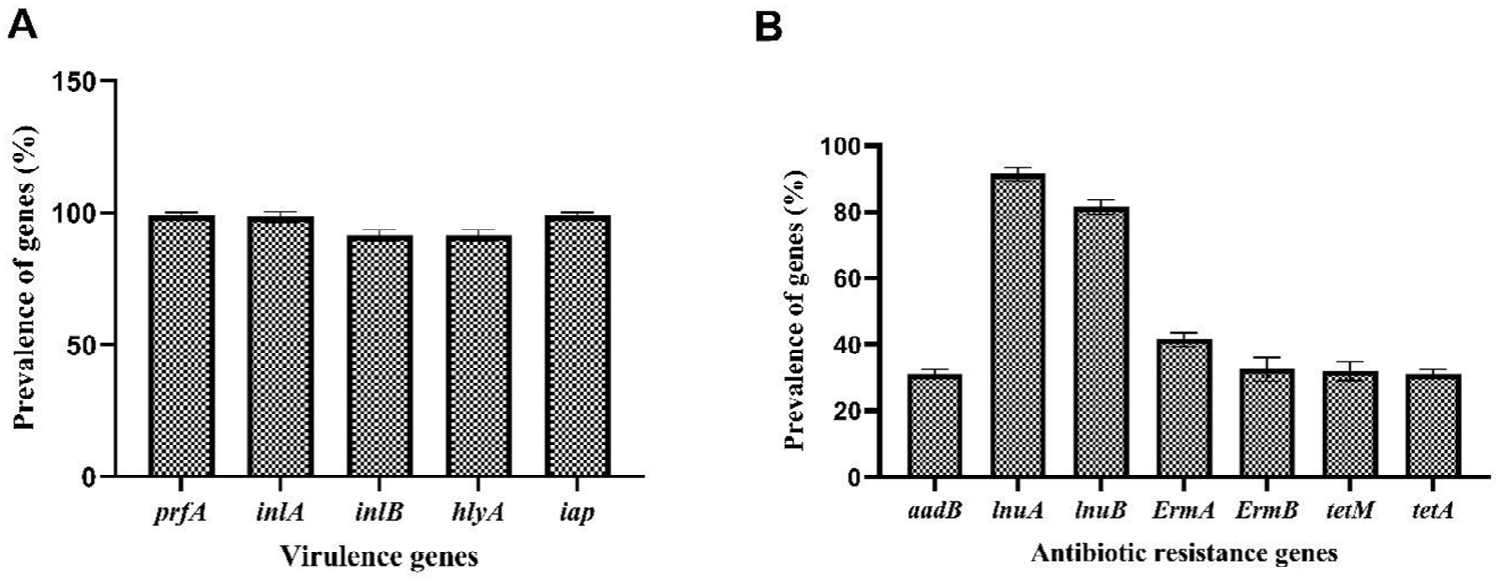
Assessment of prevalence of (A) virulence and (B) antibiotic resistance genes among *L. monocytogenes* strains separated from milk and milking equipment.

## 4 Discussion

The current study offers significant insights into the presence, possible pathogenicity, and antibiotic resistance of *L. monocytogenes* along the milk supply chain in Yazd province, Iran. Despite, *L. monocytogenes* being only found in 2.7% of cases, finding it in raw milk is solid evidence that unprocessed milk is the main source of *L. monocytogenes* contamination. The lack of *L. monocytogenes* in samples from workers’ hands and gowns further validates the efficacy of pasteurisation and basic hygiene practices. Also, the prevalence of *L. monocytogenes* in pasteurised milk was 0%. This complete elimination of pathogen is directly related to the efficacy of the commercial HTST pasteurisation process. *L. monocytogenes* is non‐spore‐forming and is highly susceptible to the time‐temperature combinations of HTST, provided that strict post‐pasteurisation hygiene is maintained to prevent recontamination. Isolation of *L. monocytogenes* from milking equipment suggests that environmental contamination may facilitate the persistence and spread of this pathogen in dairy production environments.

The fact that contamination levels at raw milk collection points are high (80 percent of cooperatives were positive) indicated that hygiene is poor at the early stages of the milk supply chain. Raw milk can be contaminated by sick animals, faeces or by the soil, silage, and water. These constitute all the areas that *L. monocytogenes* can survive (Bastam et al. 2021). These findings are in line with past research that shows that lack of cleanliness of equipment used to milk and containers used to store the milk facilitate the survival of bacteria and cross‐contamination (Aliyo and Teklemariam 2022). The occurrence of the pathogenic *L. monocytogenes* in raw milk poses great danger to human health, particularly to high‐risk groups, because sometimes raw milk may be consumed directly, or it may be used to make traditional dairy items. Kayode et al. (2022) found *L. monocytogenes* in 18.46% (12 out of 65 samples) of milk and milk product samples. These show that contamination is rather common in the Eastern Cape province of South Africa (Kayode and Okoh 2022). Similarly, Noomi et al. (2021) documented a high prevalence of *L. monocytogenes* in dairy aborted cows in Salahudeen Province, with a detection rate of 28.26% in milk from aborted cows and 30% in raw market milk. These results corroborate the current investigation by validating milk as a substantial reservoir for *L. monocytogenes* and a possible pathway for pathogen transmission within the dairy chain (Noomi et al. 2021).

The antimicrobial susceptibility tests indicated that the isolates possessed an alarming trend of resistance. As expected, a high resistance rate was seen against nalidixic acid, penicillin G and cephalosporins, which aligns with the well‐documented intrinsic resistance mechanism of *L. monocytogenes* to these specific drugs. Cephalosporins are not suitable for treating listeriosis due to intrinsic and natural resistance, but the fact that resistance to the medications has occurred shows that there may have been selective pressure due to the substantial use of antimicrobials in veterinary facilities. Notably, only 20% (2/10) of isolates exceeded the critical MAR threshold of 0.2, which is characteristic of strains originating from high‐risk environments with frequent antibiotic administration. This indicates the presence of low to moderate but significant acquired multidrug resistance in dairy production.

Conversely, the fact that isolates are fully susceptible to first‐line and other alternative clinical agents such as gentamicin, amikacin, levofloxacin, vancomycin, trimethoprim ‐sulfamethoxazole and erythromycin is a positive sign. It denotes that the existing interventions against the listeriosis are not becoming ineffective in the region. Interestingly, the phenotypic resistance to tetracycline at 30% of isolates completely matched with the molecular detection of *tetA* and *tetM*. However, several resistance genes, like *ermA*, *ermB*, *lnuA* and *lnuB*, were found even in isolates that were phenotypically susceptible. This discrepancy shows a strong epidemiological argument for the existence of silent or unexpressed resistance genes in the *L. monocytogenes* genome. The phenomenon is often observed due to the lack of functional promotors, mutations within regulatory regions, or the absence of specific environmental triggers needed to induce gene expression under standard in vitro laboratory testing conditions. As a result, these phenotypically susceptible strains remain a covert public health threat, as they carry the genetic blueprint to rapidly switch to a resistant phenotype via gene activation or horizontal transfer under shifting environmental pressures. Their presence indicates that molecular surveillance and phenotypic testing need to be used in the evaluation of the risk of antibiotic resistance.

Virulence gene profiling indicated that all the isolates possess the significant pathogenicity genes *prfA*, *inlA* and *iap*, this implies that the isolates might be very virulent. The *prfA* gene is considered to be very significant in activating the *Listeria* pathogenicity island, whereas *inlA* and *inlB* are considered very significant in infecting the host cells (Lakicevic et al. 2022). The high prevalence of *hlyA*, which codes listeriolysin O, supports the fact that these strains can escape host phagosomes and lead to systemic infection (Lakicevic et al. 2022). The presence of significant virulence genes alongside various antibiotic resistance determinants indicates that the isolated isolates have the potential to induce severe disease and may further complicate treatment should resistance patterns proliferate. Significantly, while most isolates had the *hlyA* gene, 10% (1/10) lacked this important virulence factor. Atypical or less virulent *L. monocytogenes* strains are typically thought to be strains that do not have the *hlyA* gene coding LLO. A major challenge from both an ecological and an epidemiological perspective is the continuous occurrence of *hlyA*‐negative or non‐haemolytic phenotypes in the food supply chain, because the traditional phenotype‐based diagnostic methods are unable to detect them. Besides, these unusual bacteria might include other invasion mechanisms or gain virulence features via horizontal gene transfer, underlining their crucial clinical value in public health monitoring (Maury et al. 2017).

In line with the present study, Sharma et al. (2024) recorded the ubiquitous presence of major virulence‐related genes in *L. monocytogenes* isolates derived from dairy products, which included *prfA*, *inlA*, *inlB*, *iap*, *actA*, *plcB* and *hlyA*. The continued existence of such virulence factors supports the fact that food‐acquired *L. monocytogenes* strains possess significant virulence potential and therefore the significance of their detection in milk and dairy‐associated environments to public health (Sharma et al. 2024). The overall prevalence of *L. monocytogenes* in dairy products is extremely low in Romania which points to the effectiveness of the control measures enforced at the later stages of dairy processing. Although resistant isolates are rare, their results showed that a number of isolates were resistant to SXT. These results confirm the findings of the present study that *L. monocytogenes* strains isolated from dairy products can have a clinically significant resistance pattern (Nikolaou et al. 2025). Consistent with the current investigation, Su et al. (2023) documented a similar prevalence of *L. monocytogenes* in bovine milk (2.48%) in Southwest China. Furthermore, the significant prevalence of multidrug‐resistant strains and the comprehensive array of virulence and antibiotic resistance genes revealed by whole‐genome sequencing robustly corroborate our findings, suggesting that milk‐derived *L. monocytogenes* isolates exhibit substantial pathogenic and antimicrobial resistance capabilities (Su et al. 2023).

Limitations of this study: while the total sample size (n = 370) in this surveillance was sufficient, the low isolation rate resulted in only 10 confirmed *L. monocytogenes* strains. This small number of positive samples presents a limitation and makes it hard to reliably generalise the specific antimicrobial resistance percentages for the whole region. However, the detailed molecular characterisation conducted here offers important baseline epidemiological data for future large‐scale monitoring.

## 5 Conclusion

Finally, this paper showed that raw milk is the main source of potentially virulent and multidrug‐resistant *L. monocytogenes* in the dairy supply chain of the Yazd Province. The discovery of isolates carrying essential virulence genes alongside multiple antimicrobial resistance genes highlights the potential risk posed by dairy associated strains to public health. Therefore, continuous monitoring of *L. monocytogenes*, adherence to hygiene principles in raw milk production, correct and appropriate use of antibiotics in veterinary care, and regular molecular surveillance are key measures that can be taken to prevent the circulation of pathogenic and resistant strains. Strengthening these interventions will play a key role in decreasing the global burden of listeriosis and ensuring the microbiological safety of milk and dairy products.

## Author Contributions

**Mohammad Reza Heidari**: conceptualisation, investigation, methodology, writing – original draft. **Amir Shakerian**: supervision, project administration, writing – review and editing, corresponding author. **Reza Sharafati Chaleshtori**: formal analysis, resources, data curation. **Ebrahim Rahimi**: validation, visualisation, writing – review and editing. All authors have read and agreed to the published version of the manuscript.

## Funding

The authors have nothing to report.

## Ethics Statement

This study protocol did not involve any human participants or live animal subjects. All bacterial strains were isolated from dairy sources; therefore, institutional ethical approval and informed consent were not required for this study.

## Conflicts of Interest

The authors declare no conflicts of interest.

## Data Availability

The data that support the finding of this study are available from the corresponding author upon reasonable request.
